# Inositol triphosphate–triggered calcium release from the endoplasmic reticulum induces lysosome biogenesis *via* TFEB/TFE3

**DOI:** 10.1016/j.jbc.2022.101740

**Published:** 2022-02-16

**Authors:** Mouhannad Malek, Anna M. Wawrzyniak, Michael Ebner, Dmytro Puchkov, Volker Haucke

**Affiliations:** 1 Department of Molecular Pharmacology & Cell Biology, Leibniz-Forschungsinstitut für Molekulare Pharmakologie (FMP), Berlin, Germany; 2 Faculty of Biology, Chemistry and Pharmacy, Freie Universität Berlin, Berlin, Germany

**Keywords:** calcium, lysosome biogenesis, inositol triphosphate, signaling, imaging, AF, Alexa Fluor, ASAH1, *N*-acylsphingosine amidohydrolase 1, cDNA, complementary DNA, ER, endoplasmic reticulum, ERK, extracellular signal–regulated kinase, FBS, fetal bovine serum, GSDB, goat serum dilution buffer, INPP5A, inositol polyphosphate-5-phosphatase A, IP3, inositol triphosphate, IP3R, IP3 receptor, LAMP1, lysosome-associated membrane protein 1, PLC, phospholipase C, TFE3, transcription factor E3, TFEB, transcription factor EB

## Abstract

Lysosomes serve as dynamic regulators of cell and organismal physiology by integrating the degradation of macromolecules with receptor and nutrient signaling. Previous studies have established that activation of the transcription factor EB (TFEB) and transcription factor E3 (TFE3) induces the expression of lysosomal genes and proteins in signaling-inactive starved cells, that is, under conditions when activity of the master regulator of nutrient-sensing signaling mechanistic target of rapamycin complex 1 is repressed. How lysosome biogenesis is triggered in signaling-active cells is incompletely understood. Here, we identify a role for calcium release from the lumen of the endoplasmic reticulum in the control of lysosome biogenesis that is independent of mechanistic target of rapamycin complex 1. We show using functional imaging that calcium efflux from endoplasmic reticulum stores induced by inositol triphosphate accumulation upon depletion of inositol polyphosphate-5-phosphatase A, an inositol 5-phosphatase downregulated in cancer and defective in spinocerebellar ataxia, or receptor-mediated phospholipase C activation leads to the induction of lysosome biogenesis. This mechanism involves calcineurin and the nuclear translocation and elevated transcriptional activity of TFEB/TFE3. Our findings reveal a crucial function for inositol polyphosphate-5-phosphatase A–mediated triphosphate hydrolysis in the control of lysosome biogenesis *via* TFEB/TFE3, thereby contributing to our understanding how cells are able to maintain their lysosome content under conditions of active receptor and nutrient signaling.

Late endosomes and lysosomes coordinate the degradative turnover of macromolecules, for example, proteins, lipids, and defective organelles, with cell metabolism (1, 2, 3, 4) by responding to intracellular cues and extracellular signals such as insulin or growth factors (5). Lysosome dysfunction causes lysosomal storage diseases and neurodegeneration, whereas the autophagy/lysosome degradation pathway has been shown to be involved in a plethora of human disorders including cancer (6, 7). Adaptation of the lysosomal degradation route to changing environmental or internal cues is mediated in part by the coordinated expression of autophagy/lysosome pathway genes *via* the microphthalmia—transcription factor E family, most notably its founding member transcription factor EB (TFEB). While TFEB and its close relative transcription factor E3 (TFE3) are kept cytosolically inactive under steady-state conditions, a variety of stimuli including lysosome (8, 9) and osmotic stress (10), physical exercise (11), and, most importantly, starvation (12) induce the nuclear translocation and activation of TFEB and TFE3. When nutrients are present, phosphorylation of TFEB by the mechanistic target of rapamycin complex 1 (mTORC1) inhibits its activity (12). Under conditions of starvation or growth factor deprivation, mTORC1 is inactive resulting in TFEB dephosphorylation *via* a mechanism that involves lysosomal calcium and the calcium-activated phosphatase calcineurin (13). These data give rise to a model in which repression of mTORC1 signaling and lysosomal calcium signaling regulate lysosome biogenesis *via* activation of TFEB/TFE3 in starved or growth factor–deprived cells.

However, late endosomes and lysosomes have also long been known to execute key physiological functions in signaling-active fed cells, for example, in the degradative sorting and turnover of signaling receptors, in the metabolic turnover of lipids, in plasma membrane repair, and as metabolic platforms for nutrient signaling (1, 2, 3, 4). How the biogenesis of lysosomes is regulated and maintained in signaling-active cells under conditions of ample nutrient and growth factor supply is incompletely understood but may involve protein kinase–based and/or phosphatase-based regulatory mechanisms (14, 15).

In the present study, we combine genetic and pharmacological manipulations with light and EM to show that inositol triphosphate (IP_3_)-induced calcium efflux from endoplasmic reticulum (ER) stores in the absence of the IP_3_-specific inositol polyphosphate-5-phosphatase A (INPP5A) (16, 17), an enzyme downregulated in cancer and defective in spinocerebellar ataxia, triggers lysosome biogenesis *via* calcineurin-mediated activation of TFEB and TFE3 independent of cellular nutrient status monitored by mTORC1.

## Results

### Loss of the IP_3_ 5-phosphatase INPP5A increases cellular late endosome or lysosome content

**Figure 1:**
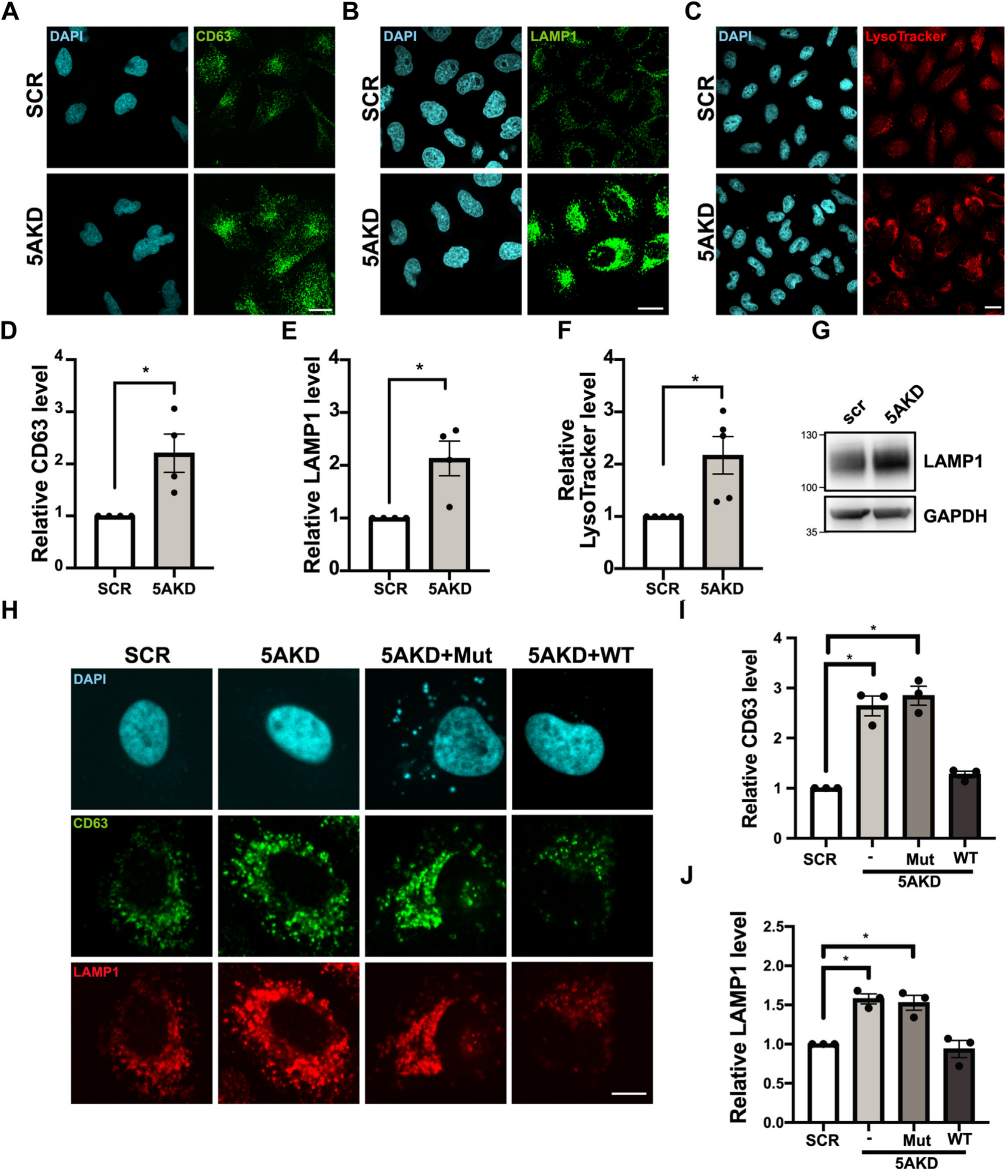
**The IP**_**3**_**5-phosphatase activity of INPP5A controls cellular late endosome and lysosome content.***A* and *B*, representative confocal images of fixed HeLa cells treated with control (SCR) or INPP5A siRNA (5AKD) and stained with specific antibodies for CD63 (*green*, *A*) or LAMP1 (*green*, *B*), respectively. *Blue*, DAPI-stained nuclei. The scale bar represents 25 μm. *C*, representative confocal images of HeLa cells treated with control (SCR) or INPP5A siRNA (5AKD) and incubated with LysoTracker (*red*) for 30 min before fixation with 4% PFA. *Blue*, DAPI-stained nuclei. The scale bar represents 25 μm. *D*–*F*, quantification of representative data shown in (*A*–*C*), respectively. Data for SCR-control siRNA-treated cells were set to 1. One-sample Student's *t* test for each pair. CD63: *p* = 0.0464, *t* = 3.282, and df = 3. LAMP1: *p* = 0.416, *t* = 3.427, and df = 3. LysoTracker: *p* = 0.031, *t* = 3.261, and df = 4. *G*, immunoblot analysis of lysates from HeLa cells treated with control (SCR) or INPP5A siRNA (5AKD). Blots were decorated with specific antibodies for LAMP1 and GAPDH as a loading control. *H*, representative confocal images of fixed HeLa cells treated with control (SCR) or INPP5A siRNA (5AKD) transfected alone or together with plasmids encoding siRNA-resistant active WT or phosphatase-defective inactive mutant (Mut) INPP5A and stained for CD63 (*green*) or LAMP1 (*red*), respectively. *Blue*, DAPI-stained nuclei. The scale bar represents 10 μm. *I* and *J*, quantification of representative data shown in *H*. Data for SCR-control siRNA-treated cells were set to 1. One-sample Student's *t* test followed by Benjamini–Hochberg correction. CD63: -/5AKD: *p* = 0.014, *t* = 8.352, and df = 2; Mut/5AKD: *p* = 0.0103, *t* = 9.777, and df = 2; WT/5AKD: *p* = 0.0550, *t* = 4.085, and df = 2. LAMP1: -/5AKD: *p* = 0.012, *t* = 9.031, and df = 2; Mut/5AKD: *p* = 0.031, *t* = 5.548, and df = 2; WT/5AKD: *p* = 0.6208, *t* = 0.5795, and df = 2. Data represent n = 4 independent experiments for CD63 and LAMP1 and five independent experiments for LysoTracker and n = 3 for rescue experiments with WT or Mut INPP5A. DAPI, 4′,6-diamidino-2-phenylindole; INPP5A, inositol polyphosphate-5-phosphatase A; IP_3_, inositol triphosphate; LAMP1, lysosome-associated membrane protein 1; PFA, paraformaldehyde.

Recent work has shown that loss or depletion of the IP_3_-specific inositol 5-phosphatase INPP5A, an enzyme downregulated in cancer (18, 19) and spinocerebellar ataxia (20), increases the intracellular levels of IP_3_ (21). IP_3_ by activating IP_3_ receptor (IP_3_R) channels in the ER causes the release of calcium into the cytoplasm. Given the multiple effects of calcium on cell signaling and intracellular membrane dynamics including the formation or dissociation of membrane contact sites between organelles (21, 22, 23), we analyzed whether loss of INPP5A might alter subcellular organelle distribution or levels. To our surprise, we found that cellular depletion of endogenous INPP5A by specific SMARTpool siRNAs (Fig. S1*A*) and the concomitant rise in IP_3_ (21) led to the accumulation of late endosomes and lysosomes containing CD63 (Fig. 1, *A* and *D*) or lysosome-associated membrane protein 1 (LAMP1) (Fig. 1, *B* and *E*). Labeling of acidified late endosomes or lysosomes with LysoTracker (Fig. 1, *C* and *F*) or immunoblotting for LAMP1 (Fig. 1*G*) confirmed their elevation in HeLa cells depleted of INPP5A. In contrast, no overt changes in the levels or distribution of early endosomal antigen 1, a marker for early endosomes (Fig. S1, *B* and *F*), the luminal ER chaperone calreticulin (Fig. S1, *C* and *G*), or β1-integrin, an adhesion receptor mostly present at the plasma membrane (Fig. S1, *D* and *H*), were observed. The *cis*-Golgi labeled by antibodies against the Golgin GM130 appeared more compact in INPP5A-depleted cells (Fig. S1, *E* and *I*) as a result of altered lipid flux across membrane contacts between the ER and the Golgi complex (21). Increased cellular content of LAMP1-containing and CD63-containing late endosomes or lysosomes in INPP5A-depleted cells was rescued by re-expression of active WT but not by catalytically inactive mutant (Mut) INPP5A (Fig. 1, *H*–*J*). Thus, the 5-phosphatase INPP5A regulates cellular late endosome and lysosome contents by controlling IP_3_ turnover.

**Figure 2:**
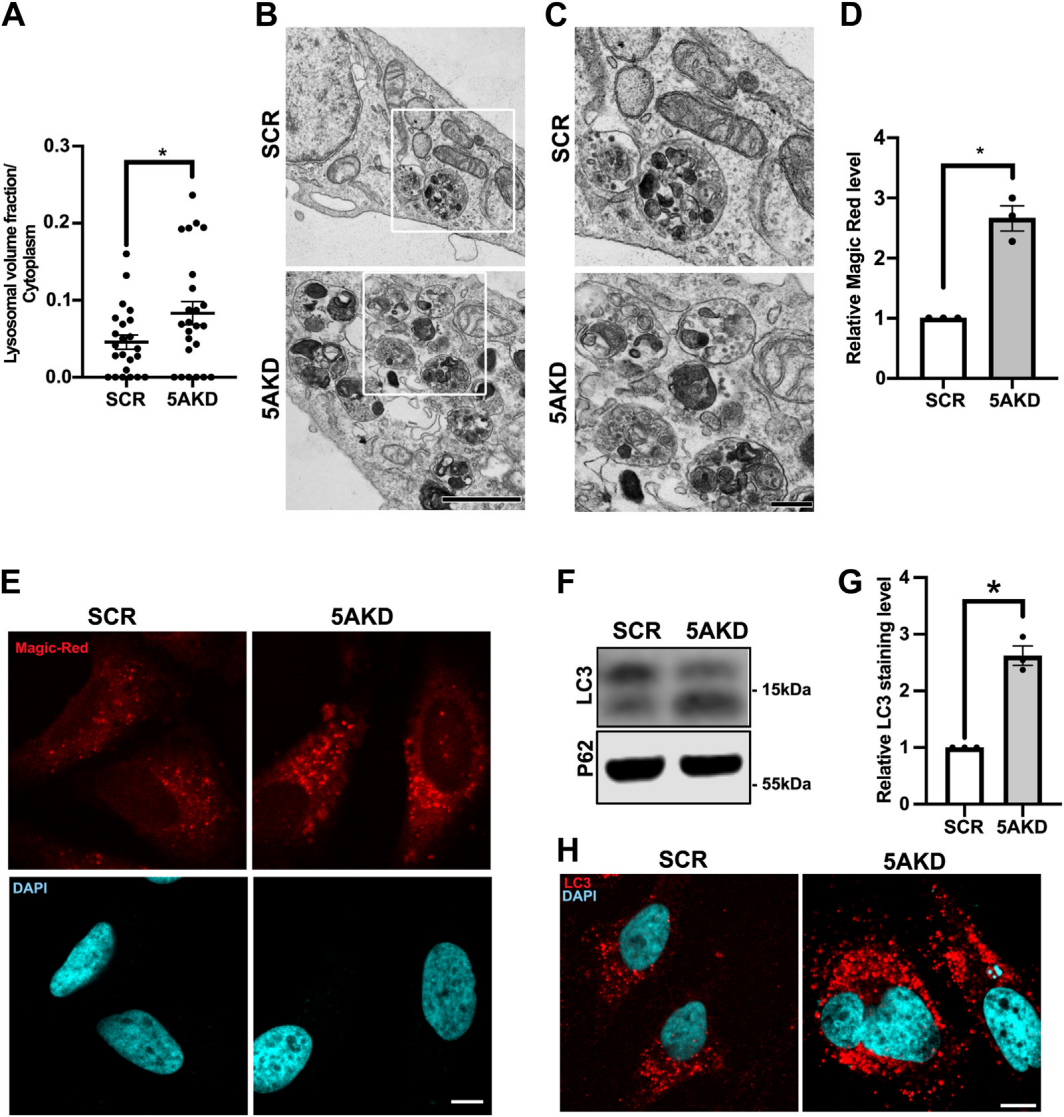
**Loss of INPP5A increases the lysosomal volume fraction and lysosomal proteolytic capacity.***A*, quantification of the lysosomal volume fraction normalized to the cytoplasm of HeLa cells treated with control (SCR) or INPP5A siRNA (5AKD). Unpaired *t* test. *p* = 0.0408, *t* = 2.106, and df = 45. *B*, representative electron micrographs of HeLa cells treated with control (SCR) or INPP5A siRNA (5AKD). Note the accumulation of degradative lysosomal organelles in 5AKD cells. The scale bar represents 1 μm. *C*, zoomed area of *white boxes* in *B*. The scale bar represents 250 nm. *D*, quantification of cathepsin L Magic Red activity in HeLa cells treated with control (SCR) or INPP5A siRNA (5AKD) one-sample *t* test. *p* = 0.0155, *t* = 7.95, and df = 2. *E*, representative confocal images of *D*. *Blue*, DAPI-stained nuclei. The scale bar represents 10 μm. *F*, representative immunoblot analysis of HeLa cells treated with control (SCR) or INPP5A siRNA (5AKD). Immunoblots were decorated with antibodies against LC3 and p62. *G*, quantification of LC3 puncta in HeLa cells treated with control (SCR) or INPP5A siRNA (5AKD) one-sample *t* test. *p* = 0.0110, *t* = 9.440, and df = 2. *H*, representative confocal images of *G*. *Blue*, DAPI-stained nuclei. The scale bar represents 10 μm. Data represent n = 23 cells for SCR and n = 24 cells for 5AKD in EM; three independent experiments for cathepsin L Magic Red and LC3 staining. DAPI, 4′,6-diamidino-2-phenylindole; INPP5A, inositol polyphosphate-5-phosphatase A.

We challenged these results from light microscopy by analyzing the lysosomal volume fraction. Ultrastructural analysis by EM and morphometry confirmed the elevated cellular content of degradative organelles in INPP5A-depleted cells (Fig. 2, *A* and *B*). Closer inspection of these samples revealed a prominent induction of multilamellar as well as electron dense late-stage lysosomal profiles (Fig. 2, *B* and *C*). The observed increase in the number of degradative organelles was paralleled by elevated proteolytic activity monitored by cathepsin magic red (Fig. 2, *D* and *E*). INPP5A-depleted cells also displayed increased levels of active LC3-II and in the number of LC3-positive autophagosomes (Fig. 2, *F*–*H*). The steady-state levels of p62, an autophagy adaptor for the clearance of aggregated proteins and a well-known substrate for autophagy/lysosome-mediated proteolysis (1), remained unchanged (Fig. 2*F*), indicating that the elevated number of autophagosomes does not result from impaired autophagic flux but from elevated biogenesis as shown later.

Collectively, these findings indicate that loss of active INPP5A leads to an increase in the cellular content of degradative lysosomes and autophagosomes but not of other organelles.

### Accumulation of late endosomes or lysosomes upon depletion of INPP5A is not the result of altered nutrient signaling, lysosomal calcium release or the induction of the ER stress response

Lysosome biogenesis is repressed by the mTORC1-dependent phosphorylation and sequestration of TFEB in fed cells. Hence, suppression of mTORC1 activity could lead to decreased TFEB phosphorylation and, thereby, increase TFEB nuclear translocation to induce lysosome biogenesis in the absence of INPP5A. To determine whether reduced mTORC1 signaling might contribute to elevated cellular lysosome content in INPP5A-depleted cells, we analyzed the levels of phosphorylated S6 kinase 1, a direct substrate of mTORC1 (5). Loss of INPP5A did not alter the levels of phosphorylated S6 kinase 1 normalized to total S6K1 (Fig. S2, *A* and *B*). Recent studies show that in addition to mTOR, TFEB may also be phosphorylated by other kinases, most notably extracellular signal–regulated kinase (ERK) (15, 24). However, no overt change in the activity status of ERK was detectable in INPP5A-depleted cells (Fig. S2*C*).

Given that INPP5A acts on IP_3_Rs in the ER (21), we considered the possibility that the observed accumulation of lysosomes upon depletion of INPP5A might be an indirect consequence of ER stress, which in turn could target the ER for autophagic turnover in lysosomes. However, INPP5A knockdown cells did not display elevated levels of phosphorylated protein kinase R–like endoplasmic reticulum kinase (Fig. S3, *A* and *C*), a mediator of ER stress that has been shown to induce lysosome biogenesis (25) or the ER stress–inducible chaperone binding immunoglobulin protein (Fig. S3, *A* and *B*).

Given that the ER plays a role in the refilling of lysosomal calcium stores (26) and calcium release from the lysosome lumen *via* mucolipin 1 can trigger TFEB activation in starved cells (13), we considered a possible function for mucolipin 1 as a downstream element in the cascade that triggers lysosome accumulation in INPP5A-depleted cells. We tested this possibility by codepleting HeLa cells of mucolipin 1 and INPP5A. However, loss of mucolipin 1 (Fig. S4*C*) further aggravated the accumulation of lysosomes in cells depleted of INPP5A (Fig. S4, *A* and *B*), possibly as a result of altered lysosomal calcium buffering capacity. These data are further consistent with the observation that lysosomal calcium release *via* mucolipin 1 and the subsequent induction of lysosome biogenesis reflect a starvation-induced response that does not occur in fed signaling-active cells (13).

We conclude that lysosome accumulation in INPP5A-depleted cells is not the result of reduced mTORC1 signaling, induction of the ER stress response, or lysosomal calcium release *via* mucolipin 1.

### IP_3_-induced calcium release activates calcineurin to increase cellular lysosome content downstream of receptor signaling *via* phospholipase C

**Figure 3:**
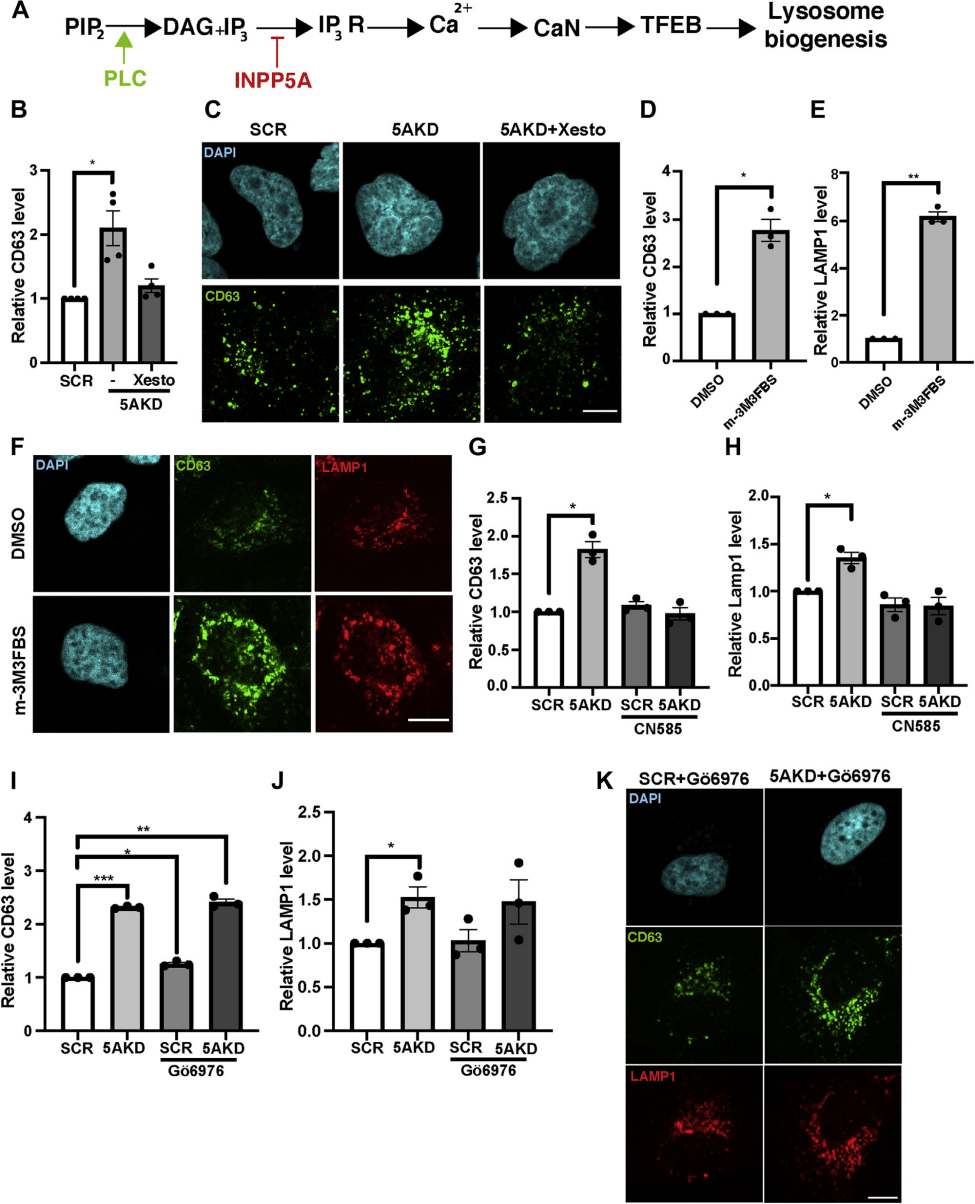
**IP**_**3**_**-induced calcium release increases cellular lysosome content downstream of receptor signaling *via* phospholipase C and Ca**^**2+**^**/calcineurin.***A*, schematic representation of phospholipase C (PLC)-mediated cleavage of phosphatidylinositol 4,5-bisphosphate (PIP_2_) into diacylglycerol (DAG) and IP_3_ downstream of receptor signaling. IP_3_ triggers Ca^2+^ release from the ER *via* IP_3_ receptors (IP_3_Rs), a pathway repressed by INPP5A-mediated IP_3_ hydrolysis. Ca^2+^ release activates calcineurin (CaN), which dephosphorylates and thereby activates TFEB to induce lysosomal gene expression. *B*, quantification of relative CD63 levels in representative data shown in *C*. Data for SCR-control siRNA-treated cells were set to 1. One-sample Student's *t* test followed by Benjamini–Hochberg correction. -/5AKD: *p* = 0.0267, *t* = 4.072, and df = 3. Xesto/5AKD: *p* = 0.1660, *t* = 1.822, and df = 3. *C*, representative confocal images of fixed HeLa cells treated with control (SCR) or INPP5A siRNA (5AKD) treated with DMSO (−) or with 1 μM xestospongin C (Xesto) for 18 h and stained for CD63 (*green*). *Blue*, DAPI-stained nuclei. The scale bar represents 10 μm. *D* and *E*, quantification of representative data shown in *F*. Data for SCR-control siRNA-treated cells were set to 1. One-sample Student's *t* test. CD63/m-3M3FBS: *p* = 0.0173, *t* = 7.506, and df = 2. LAMP1/m-3M3FBS: *p* = 0.0017, *t* = 24.14, and df = 2. *F*, representative confocal images of fixed HeLa cells treated with DMSO or with 1 μM m-3M3FBS for 24 h and stained with specific antibodies for CD63 (*green*) and LAMP1 (*red*). *Blue*, DAPI-stained nuclei. The scale bar represents 10 μm. Zoomed views of low magnification images shown in Fig. S5*B* are shown. *G* and *H*, quantification of relative levels of CD63 (*G*) and LAMP1 (*H*), in HeLa cells treated with DMSO or with 5 μM CN585 for 24 h. Data for SCR-control siRNA-treated cells were set to 1. One-sample Student's *t* test followed by Benjamini–Hochberg correction. CD63/5AKD: *p* = 0.0154, *t* = 7.968, and df = 2. CD63/SCR + CN585: *p* = 0.2318, *t* = 1.697, and df = 2. CD63/5AKD + CN585: *p* = 0.7773, *t* = 0.3231, and df = 2. LAMP1/5AKD: *p* = 0.0287, *t* = 5.774, and df = 2. LAMP1/SCR + CN585: *p* = 0.182, *t* = 2.011, and df = 2. LAMP1/5AKD + CN585: *p* = 0.2254, *t* = 1.732, and df = 2. *I* and *J*, quantification of relative levels of CD63 (*I*) and LAMP1 (*J*) in HeLa cells treated with control (SCR) or INPP5A siRNA (5AKD) and treated with DMSO or 100 nM CN585 for 24 h. Data for DMSO-treated SCR-control siRNA-treated cells were set to 1. One-sample Student’s *t* test followed by Benjamini–Hochberg correction. CD63/5AKD: *p* = 0.0001, t = 90.39, and df = 2. CD63/SCR + Gö6976: *p* = 0.0111, *t* = 9.407, and df = 2. CD63/5AKD + Gö6976: *p* = 0.0017, *t* = 24.32, and df = 2. LAMP1/5AKD: *p* = 0.0497, *t* = 4.317, and df = 2. LAMP1/SCR + Gö6976: *p* = 0.8347, *t* = 0.2369, and df = 2. LAMP1/5AKD + Gö6976: *p* = 0.2036, *t* = 1.863, and df = 2. *K*, representative confocal images of fixed HeLa cells treated with control (SCR) or INPP5A siRNA (5AKD) and with or without 100 nM Gö6976 (24 h) and stained with specific antibodies against CD63 (*green*) or LAMP1 (*red*). *Blue*, DAPI-stained nuclei. The scale bar represents 10 μm. Data represent n = 4 independent experiments for xestospongin C treatment, n = 3 for treatments with m-3M3FBS, CN585, and Gö6976. DAPI, 4′,6-diamidino-2-phenylindole; DMSO, dimethyl sulfoxide; ER, endoplasmic reticulum; INPP5A, inositol polyphosphate-5-phosphatase A; IP_3_, inositol triphosphate; LAMP1, lysosome-associated membrane protein 1; TFEB, transcription factor EB.

Based on these negative data, we considered an alternative scenario according to which INPP5A by hydrolyzing soluble IP_3_ constitutes a negative regulatory element in the signaling cascade that triggers IP_3_R-mediated calcium release from the ER lumen (27). In this cascade phospholipase C (PLC) catalyzes cleavage of phosphatidylinositol 4,5-bisphosphate into diacylglycerol and IP_3_ downstream of receptor activation in fed signaling-active cells (Fig. 3*A*). We therefore hypothesized that elevated IP_3_ levels in the absence of INPP5A might activate TFEB/TFE3 function, and, thereby, induce lysosome biogenesis. We tested the putative role of IP_3_ in this pathway at multiple levels. First, we applied xestospongin C, a blocker of IP_3_R-mediated calcium release from the ER in HeLa cells depleted of INPP5A. Xestospongin C application potently rescued the accumulation of CD63-positive late endosomes or lysosomes in INPP5A-depleted cells (Figs. 3, *B* and *C* and S5*A*). Conversely, we tested the consequences of hyperactivation of PLC, that is, the enzyme that generates IP_3_ downstream of receptor signaling in genetically unperturbed WT HeLa cells. Pharmacological hyperactivation of PLC by m-3M3FBS sufficed to induce lysosome biogenesis as evidenced by elevated cellular content of CD63- and LAMP1-containing organelles (Figs. 3, *D*–*F* and S5*B*). These data establish that activation of PLC downstream of receptor signaling can trigger lysosome biogenesis *via* a pathway that requires IP_3_R-mediated calcium release from the ER (Fig. 3*A*).

Next, we wanted to dissect how IP_3_R-mediated calcium release triggers lysosome biogenesis downstream of receptor-induced PLC signaling. Elevated cytosolic calcium levels are known to activate the calcium-regulated protein phosphatase calcineurin, which in turn can induce lysosome biogenesis by dephosphorylating and, thereby, activating TFEB and TFE3 (13). PLC may conceivably act *via* an alternative route involving the generation of diacylglycerol, which together with the IP_3_-induced calcium release could activate protein kinase C, another enzyme that has been suggested to induce TFEB activation (14). We probed these alternative mechanisms pharmacologically. Treatment of INPP5A-depleted HeLa cells with CN585, a specific inhibitor of cellular calcineurin activity (28), completely reversed the observed increase in late endosome (*i.e.*, CD63) and lysosome (*i.e.*, LAMP1) content (Figs. 3, *F*–*H* and S5*C*). By contrast, inhibition of PKC activity in the presence of Gö6976 (29) failed to revert the elevation in late endosome/lysosome content induced by loss of INPP5A (Fig. 3, *I*–*K*).

These results show that PLC activation downstream of receptor signaling triggers lysosome biogenesis *via* IP_3_-induced calcium release and activation of calcineurin.

### IP_3_-induced calcium release from internal stores induces lysosome biogenesis *via* activation of TFEB/TFE3 and induction of lysosomal gene expression

**Figure 4:**
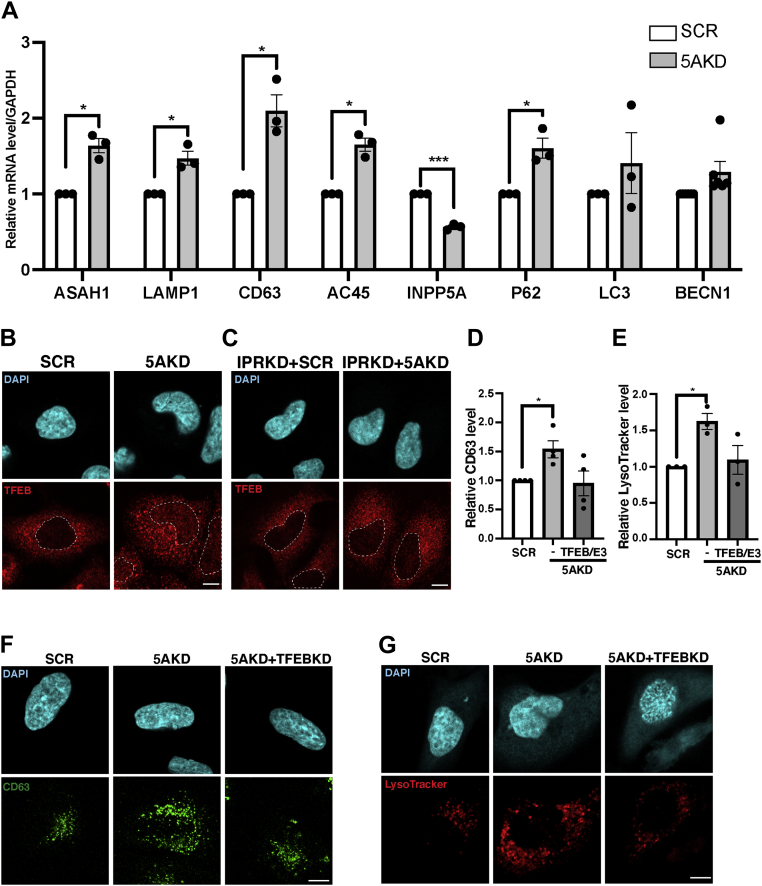
**Loss of INPP5A induces lysosomal gene expression *via* nuclear translocation of TFEB/TFE3.***A*, relative mRNA levels of *INPP5A* and different TEFB-targeted gene: *ASAH1*, *LAMP1*, *CD63*, *AC45*, *P62*, *LC3*, and *BCN1* in HeLa cells treated with scrambled control siRNA control (SCR) or siRNA against INPP5A (5AKD). *B* and *C*, representative confocal images of HeLa cells treated with control (SCR) or INPP5A siRNA (5AKD) either alone (*B*) or in combination with siRNA against IPRs (IPRKD) (*C*). Cells were stained postfixation with specific antibodies against TFEB (*red*). *Blue*, DAPI-stained nuclei. The scale bar represents 10 μm. *Dotted white line* delimits the nucleus in all images. *D* and *E*, quantification of representative data shown in (*F* and *G*), respectively. Data for SCR-control siRNA-treated cells were set to 1. One-sample Student's *t* test followed by Benjamini–Hochberg correction. CD63: *p* = 0.034, *t* = 3.711, and df = 3. LysoTracker: *p* = 0.0292, *t* = 5.719, and df = 2. *F* and *G*, representative confocal images of HeLa cells treated with control (SCR) or INPP5A siRNA (5AKD) either alone or in combination with siRNA against TFEB and TFE3 (TFEB/E3) and stained with antibodies against CD63 (*green*, *F*) or incubated with LysoTracker (*red*) for 30 min prior to PFA fixation (*red*, *G*), respectively. *Blue*, DAPI-stained nuclei. The scale bar represents 10 μm. All data represent three independent experiments except for BCN1 (n = 6) and CD63/TFEB–E3 (n = 4). *ASAH1*, *N*-acylsphingosine amidohydrolase 1; DAPI, 4′,6-diamidino-2-phenylindole; INPP5A, inositol polyphosphate-5-phosphatase A; *LAMP1*, lysosome-associated membrane protein 1; PFA, paraformaldehyde; TFE3, transcription factor E3; TFEB, transcription factor EB.

Previous work (13) has demonstrated that calcium-activated calcineurin triggers the nuclear translocation of TFEB (and its close cousin TFE3), which in turn induces late endosomal/lysosomal gene expression (compare Fig. 3*A*). Consistent with this mechanism, we found the mRNA expression levels of lysosomal genes including lysosomal acid ceramidase (ASAH1; *N*-acylsphingosine amidohydrolase 1), LAMP1, CD63, and the vATPase (monitored by the ATP6AP1 gene encoding its Ac45 subunit) to be significantly increased in INPP5A knockdown cells (Fig. 4*A*). The expression of autophagy genes including the autophagy adaptor p62, LC3, and beclin 1, a component of the VPS34 lipid kinase complex required for autophagy, also appeared to be modestly elevated, although with the exception of p62 did not rise to statistical significance (Fig. 4*A*). The observed induction of lysosomal gene expression in INPP5A-depleted cells was paralleled by the redistribution of TFEB to the nucleus of cells (Fig. 4*B*), a phenotype that was rescued by blockade of IP_3_-induced calcium efflux in the absence of IP_3_Rs (Fig. 4*C*). Finally, we asked whether activation and nuclear translocation of TFEB (and likely TFE3) in the absence of INPP5A indeed underlies elevated lysosome biogenesis in INPP5A-depleted cells. Strikingly, codepletion of TFEB and TFE3 prevented the accumulation of CD63-containing late endosomes/lysosomes in INPP5A knockdown cells (Fig. 4, *D* and *F*). Similar results were seen if late endosomes or lysosomes were labeled with LysoTracker (Fig. 4, *E* and *G*). These results show that IP_3_-induced calcium release from internal stores in the absence of INPP5A induces lysosome biogenesis *via* activation of TFEB/TFE3.

In summary, our data unravel an mTORC1-independent pathway for the control of lysosome biogenesis that involves the PLC-mediated generation of IP_3_, which is counteracted by INPP5A, IP_3_-induced calcium release from ER stores, and the induction of lysosomal gene expression *via* TFEB/TFE3 activation and nuclear translocation by calcineurin.

## Discussion

In this study, we identify an mTORC1-, ERK-, and PKC-independent pathway for the regulation of lysosome biogenesis *via* IP_3_-induced calcium release from ER stores downstream of receptor signaling. Multiple lines of evidence support this scenario. First, we demonstrate that elevation of IP_3_ by interfering with its hydrolysis *via* the IP_3_-specific 5-phosphatase INPP5A triggers the activation and nuclear translocation of TFEB resulting in the induction of late endosomal/lysosomal gene and protein expression in the absence of any overt alteration in mTORC1 signaling. Mucolipin 1 is dispensable for this pathway (compare Fig. S4). Second, genetic or pharmacological interference with IP_3_R function or loss of TFEB/TFE3 rescues the observed increase in cellular content of late endosomes and lysosomes in INPP5A-depleted cells. Third, we show that hyperactivation of PLC, which generates IP_3_ downstream of receptor signaling in genetically unperturbed cells, phenocopies loss of INPP5A with respect to the increase in cellular lysosome content. Importantly, the mechanism we describe here is distinct from the previously observed role of the integrated ER stress response in TFEB/TFE3 activation that depends on the phosphorylation and resulting activation of the ER stress–regulated protein kinase R–like endoplasmic reticulum kinase (25). It is also independent of mucolipin 1-induced calcium release from the lysosome lumen, a mechanism that selectively triggers lysosome biogenesis in starved cells (13). All these mechanisms, however, share a requirement for elevated cytosolic calcium levels that reflects the involvement of calcineurin in TFEB/TFE3 activation (13). At present, we cannot rule out that additional signaling factors beyond calcium/calcineurin contribute to the IP_3_-induced transcriptional activation of lysosome biogenesis *via* TFEB/TFE3. Although not supported by our current data (compare Fig. 3), it remains possible that under some conditions and/or in specific cell types, the PLC-dependent IP_3_-triggered calcineurin pathway for TFEB/TFE3 activation synergizes with the previously described PKC-based mechanism for TFEB activation (14). Our work thus extends the known physiological stimuli that can trigger TFEB/TFE3-induced lysosome biogenesis to signaling *via* PLC-linked receptors that impinge on IP_3_-mediated regulation of ER calcium stores. These include many G protein–coupled receptors including not only α1-adrenergic, H1 histamine, and metabotropic glutamate receptors but also tyrosine kinase receptors such as fibroblast growth factor receptor 1, the epidermal growth factor receptor, or lymphocyte antigen receptors (30, 31). Consistent with our findings, very recent work suggests that resveratrol, a natural active substance found in fruits and nuts and thought to counteract aging, promotes TFEB-mediated lysosome biogenesis *via* calcium release from the ER (32).

Moreover, our data together with previous studies demonstrate that multiple calcium sources may contribute to TFEB activation including the lumen of lysosomes (13), extracellular calcium influx *via* sodium/calcium exchange (10), and calcium efflux from the lumen of the ER (this study). The multiplicity of calcium sources involved in TFEB/TFE3 regulation may reflect the complexity of the physiological triggers that impinge on cellular lysosome functions in different cells and tissues (8, 9) and opens the possibility for complex patterns of feedback regulation and synergism.

Finally, our data may help to explain the benefits of depletion of INPP5A in cancers such as squamous cell carcinoma (18, 19). Loss of INPP5A in cancer cells may help to fuel cell growth and division under restricted supply of nutrients by promoting the lysosome-mediated degradation of proteins internalized *via* macropinocytosis into amino acids (33), which in turn serve to sustain mTORC1 activity. Future studies will be needed to address this possibility in detail.

## Experimental procedures

### Cell culture

HeLa cells were obtained from the American Type Culture Collection. Cells were cultured in Dulbecco's modified Eagle's medium with 4.5 g/l^−1^ glucose (Lonza) containing 10% heat-inactivated fetal bovine serum (FBS) (Gibco, Inc) and 100 U ml^−1^ penicillin and 100 μg ml^−1^ streptomycin (Gibco, Inc) during experimental procedures. Cells were not used beyond passage 30. All cell lines were routinely tested for mycoplasma contamination on a monthly basis.

### Chemicals and inhibitors

All chemicals and inhibitors were dissolved according to the manufacturer's instructions to indicated concentrations at stock solutions. Working concentrations are indicated for each experiment. m–3M3FBS was prepared at 10 mM stock solution and purchased from Sigma–Aldrich (catalog no.: 525185). Xestospongin C prepared at 1 mM stock solution and purchased from Abcam (catalog no.: ab120914). LysoTracker Red DND-99 purchased from Thermo Fisher Scientific at 1 mM. Gö6976 purchased from Sigma (catalog no.: 365250-500UG) and prepared at 1 mM. Calcineurin inhibitor VIII, CN585, was purchased from Calbiochem (CAS 1213234-31-1) and prepared at 1 mM.

### Antibodies

Antibodies used in this study are listed in Table S2. Secondary antibodies for immunoblotting were either peroxidase-conjugated to a fluorescent dye for fluorescence detection. Secondary antibodies for immunocytochemistry were Alexa Fluor (AF) conjugated. ICC—immunocytochemistry; IB—immunoblotting; rb—rabbit, ms—mouse, gt—goat; dk—donkey; AF; α—anti; and H & L—heavy and light chain.

### Plasmids

We used the following plasmids or derivatives thereof: INPP5A WT and phosphatase-deficient mutant-encoding plasmids were designed with GeneArt services from Thermo Fisher Scientific. INPP5A complementary DNAs (cDNAs) were inserted into the EcoRV/XbaI sites of pcDNA3.1(+) and verified by dsDNA sequencing.

### siRNAs and oligonucleotides

siRNA oligonucleotides used in this study (single or SMARTpools consisting of four siRNAs) are listed in Table S1.

### Quantitative real-time RT–PCR

Cultures were harvested, and total RNA was extracted using an RNeasy Mini kit (QIAGEN). The concentration of RNA was measured with a spectrophotometer (Nano Drop), and 500 ng of RNA per sample were used in each reaction according to instruction in SuperScript IV First-Strand Synthesis System (Thermo Fisher Scientific) to create cDNA library. Hundred nanograms of cDNA were used in each reaction according to SsoAdvanced Universal SYBR Green Supermix (QIAGEN). Samples were subsequently loaded on to StepOnePlus Real-Time PCR System (Thermo Fisher Scientific). Ct values were obtained and converted to relative mRNA expression levels. GAPDH was used as a reference/normalization control. Primer sequences for quantitative PCR are listed in Table S1.

### siRNA and plasmid transfection

About 100,000 (HeLa) cells, with low passage numbers, are reverse transfected using jetPRIME transfection reagent according to the manufacturer protocol using 100 nM of each siRNA. If DNA cotransfection is needed, cells are first reverse-transfected with siRNA, and 24 h later, 2 μg of DNA is transfected using the jetPRIME protocol.

### Light microscopy of cultured cells

Treated cells were washed once with PBS and fixed with 4% paraformaldehyde/4% sucrose for 20 min at room temperature. Samples were washed three times with PBS + 10 mM MgCl_2_ and blocked/permeabilized with goat serum dilution buffer (GSDB) (10% goat serum, 100 mM NaCl, 0.3% Triton X-100, in PBS) for 30 min at room temperature. Primary antibodies were diluted in GSDB and applied for 2 h at room temperature. Next, cells were washed three times with PBS + 10 mM MgCl_2_ and incubated with appropriate AF dye–conjugated secondary antibodies diluted in GSDB. Cells were washed three times with PBS–10 mM MgCl_2_, dipped in the water, and mounted with Immunomount supplemented with 4′,6-diamidino-2-phenylindole to stain nuclear DNA. Images were acquired with a minimum resolution (512px × 512px) for confocal imaging using Zeiss LSM 710 or LSM780 microscopes. Image analysis and quantification were performed using FIJI (https://imagej.net/software/fiji/).

### Cell lysates and immunoblotting

Cells were washed three times in ice-cold PBS and collected in PBS with 1% Triton X-100, 0.3% protease inhibitor cocktail (Sigma–Aldrich), and phosphatase inhibitors (cocktails 2 and 3; Sigma–Aldrich). Protein levels were quantified using Bradford reagent (Sigma–Aldrich). Equal concentration lysates in Laemmli sample buffer were boiled for 5 min; between 10 and 50 μg protein was resolved by SDS-PAGE and analyzed *via* immunoblot using LI-COR 800CW and 680RD infrared secondary antibodies as indicated.

### Labeling lysosomes with lysoTracker

To stain lysosomes, astrocytes were incubated with 250 to 500 nM of LysoTracker red or green in 1 ml of complete fresh medium for 45 min, washed three times, and imaged immediately in live-cell imaging solution (Hanks' balanced salt solution containing 5% fetal calf serum and 20 mM Hepes [pH 7.4]) using the Zeiss laser scanning confocal microscope LSM710.

### Determination of cathepsin activity

Cathepsin L activity was monitored by the Magic Red Cathepsin detection kit (Bio-Rad). HeLa cells were loaded with Magic Red Cathepsin L reagent in complete fresh Dulbecco's modified Eagle's medium for 60 min at 37 °C in the dark, washed three times, and imaged immediately in live-cell imaging solution (Hanks' balanced salt solution containing 5% fetal calf serum and 20 mM Hepes [pH 7.4]) using a Zeiss laser scanning confocal microscope LSM710.

### EM

Cells were fixed with 2% glutaraldehyde in PBS, washed, pelleted, embedded in agar, postfixed with 1% osmium tetroxide in water, dehydrated with methanol, infiltrated, and embedded in epoxy resin. Images of cell crosssections were obtained at Zeiss 900 TEM. Grid was superimposed over cell profiles in FIJI standard package for stereology-based estimation of lysosomal volume fraction in the cellular cytoplasm.

### Statistics and reproducibility

Values are depicted as mean ± SEM or mean. One-sample two-sided *t* tests were used for comparisons with control group values that had been set to one for normalization purposes and that therefore did not fulfill the requirement of two-sample *t* tests. The Benjamini–Hochberg procedure was used to correct for multiple testing based on the acceptance of a false discovery rate of 5% (see the legends to the figures). GraphPad Prism, version 8, software (GraphPad Software, Inc) was used for statistical analysis. The level of significance is indicated in the figures by asterisks (∗*p* < 0.05; ∗∗*p* < 0.01; ∗∗∗*p* < 0.001; and ∗∗∗∗*p* < 0.0001) and provided in the legends to the figures as exact *p* value obtained by the indicated statistical test. No statistical method was used to predetermine sample size as sample sizes were not chosen based on prespecified effect size. Instead, multiple independent experiments were carried out using several sample replicates as detailed in the legends to the figures.

## Data availability

All data are contained in this article and in the supporting information. Source data are available from the corresponding author (haucke@fmp-berlin.de) upon request.

## Supporting information

This article contains supporting information.

## Conflict of interest

The authors declare that they have no conflicts of interest with the contents of this article.
