# Supplementary material for: Inositol triphosphate–triggered calcium release from the endoplasmic reticulum induces lysosome biogenesis via TFEB/TFE3
Source: J Biol Chem. 2022 Feb 16;298(3):101740. doi: 10.1016/j.jbc.2022.101740 (PMC8914386; doi:10.1016/j.jbc.2022.101740)
Supplement: Supplemental Figures S1–S5 and Tables S1–S2 [file mmc1.pdf]

Supplementary Information for

Inositol triphosphate-triggered calcium release from the endoplasmic reticulum induces lysosome biogenesis via TFEB/ TFE3

Mouhannad Malek, Anna M. Wawrzyniak, Michael Ebner, Dmytro Puchkov, Volker Haucke

Inventory of supplementary items

Supplementary figures
Supplementary figure 1 | INPP5A loss does not impact on the distribution or cellular levels of the ER, early endosomes, or cell surface β1-integrins and leads to Golgi compaction.
Supplementary figure 2 | INPP5A loss does not affect mTORC1 or ERK signaling.
Supplementary figure 3 | INPP5A-depleted cells do not suffer from ER stress.
Supplementary figure 4 | Lysosome accumulation in INPP5A-depleted cells is further aggravated by loss of Mucolipin 1.
Supplementary figure 5 | Phospholipase C-mediated generation of IP_3 triggers lysosome biogenesis via activation of calcineurin.

Supplementary tables
Supplementary table 1 | Oligonucleotides used in this study
Supplementary table 2 | Antibodies used in this study

Supplementary figures

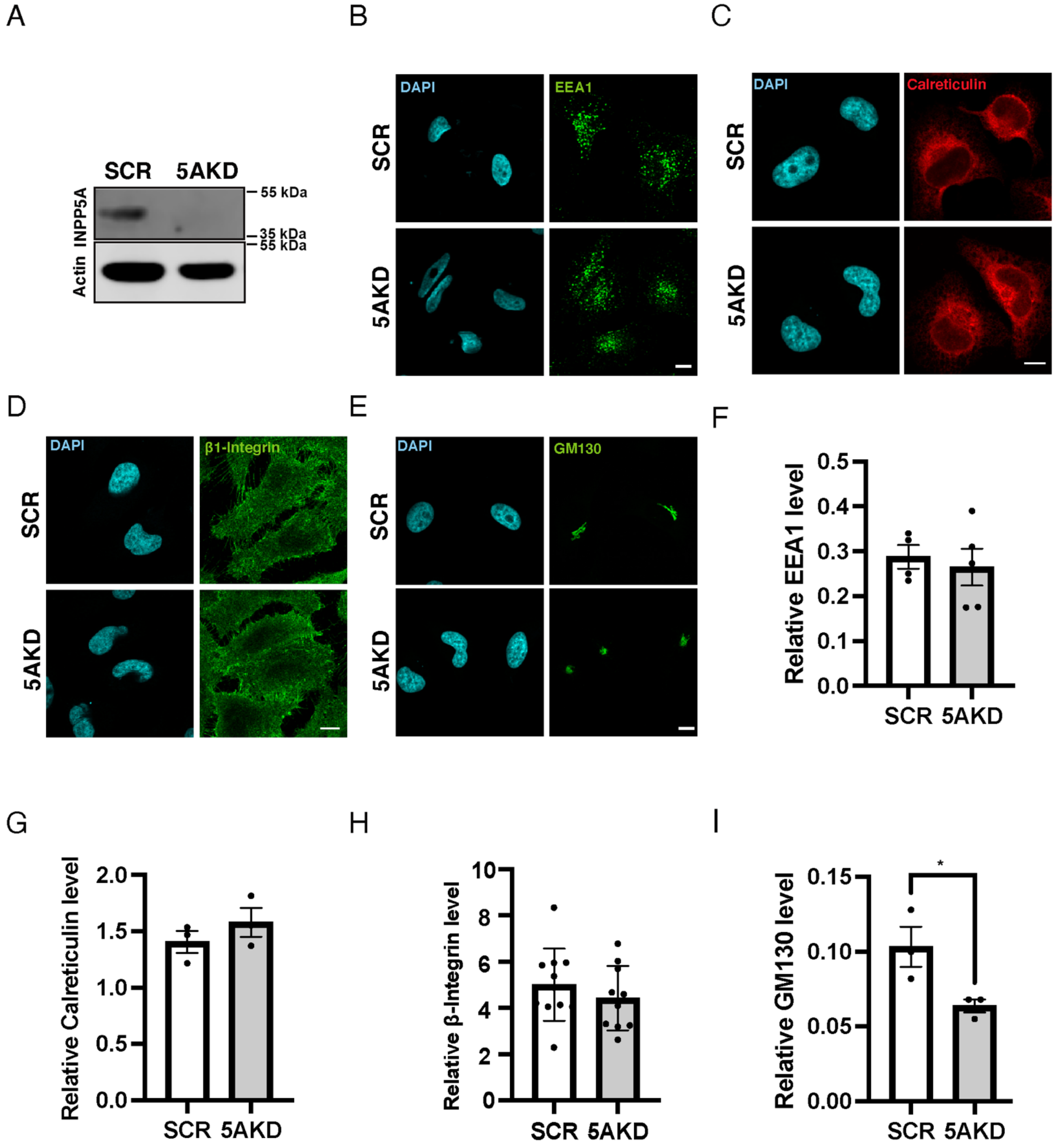

Supplementary figure 1 | INPP5A loss does not impact on the distribution or cellular levels of the ER, early endosomes, or cell surface β1-integrins and leads to Golgi compaction. (A) Representative immunoblot analysis of HeLa cells treated with control (SCR) or INPP5A siRNA (5AKD). Immunoblots were decorated with antibodies against INPP5A and actin. (B-E) Representative confocal images of fixed HeLa cells treated with control (SCR) or INPP5A siRNA (5AKD) and stained for EEA1 (green, B), calreticulin (red, C), β1-integrin (green, D), or GM130 (green, E). Blue, DAPI-stained nuclei. Scale bar, 10 µm. Note that the views in panels C and E are derived from the same experiment, in which cells were triply stained for DAPI, calreticulin (C) and GM130 (E). (F-I) Quantification of organelle levels as exemplified by representative data in B-E. Unpaired t-test: EEA1: p=0.6756, t=0.4366, df=7. Calreticulin: p=0.3461, t=1.067, df=4. GM130: p=0.484, t=2.809, df=4. β1-integrin: p=0.3785, t=0.9017, df=19. Data for EEA1 represent 5 independent experiments, for GM130 and calreticulin: n=3, β1-integrin: n=10.

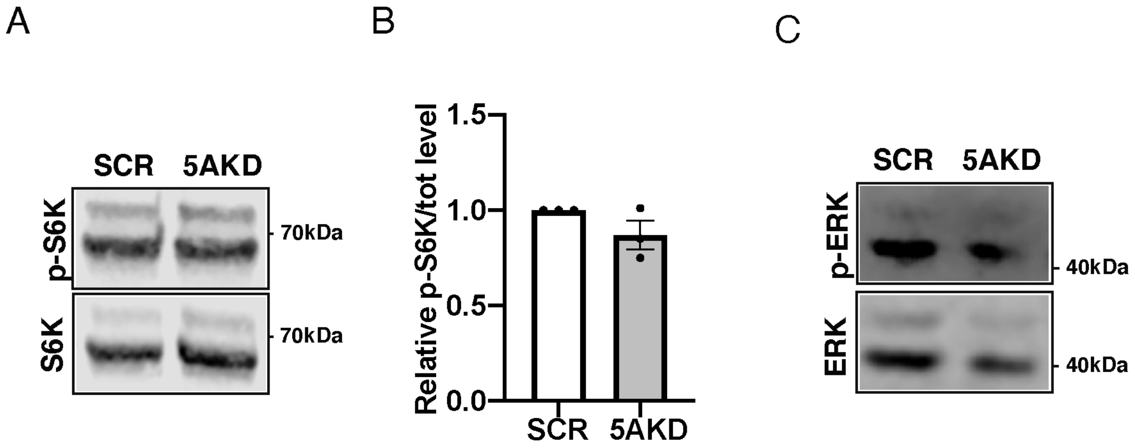

Supplementary figure 2 | INPP5A loss does not affect mTORC1 or ERK signaling. (A,C) Representative immunoblot analysis of HeLa cells treated with control (SCR) or INPP5A siRNA (5AKD). Immunoblots were decorated with antibodies against phospho-S6K1 (p-S6K) and total S6K1 (S6K), phospho-ERK(p-ERK) and total ERK (ERK). (B) Quantification of pS6K1/ total S6K1 levels as exemplified by representative shown in (A) from n=3 independent experiments. Data for SCR-control siRNA-treated cells were set to 1. One sample student's t-test p-S6K: p=0.3457, t=1.224, df=2.

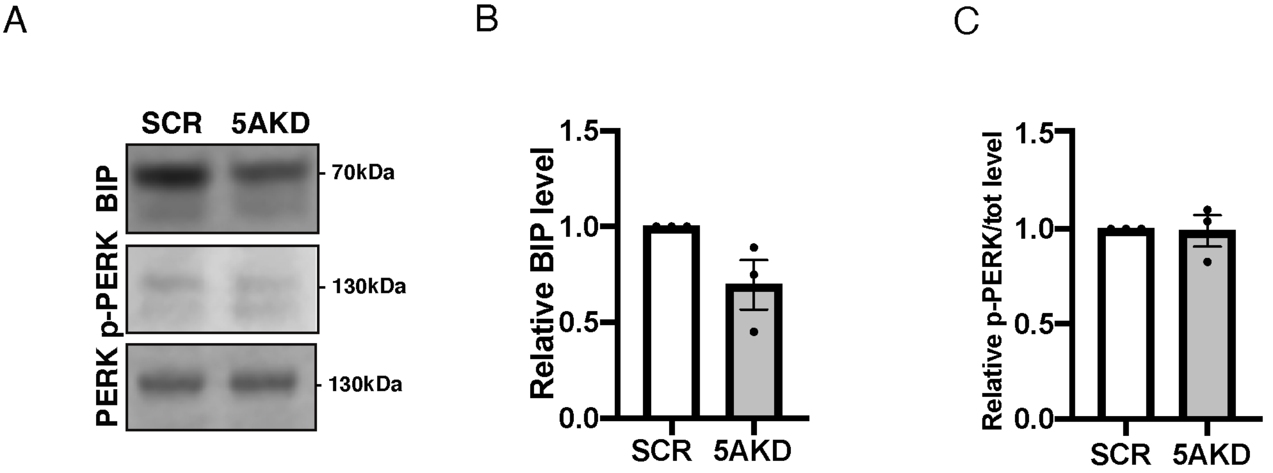

Supplementary figure 3 | INPP5A-depleted cells do not suffer from ER stress. (A) Representative immunoblot analysis of HeLa cells treated with control (SCR) or INPP5A siRNA (5AKD). Immunoblots were decorated with antibodies against BIP, phospho-PERK and total PERK. (B,C) Quantification of BIP (B) and pERK/ total ERK (C) levels as exemplified by representative data shown in (A) from n=3 independent experiments. Data for SCR-control siRNA-treated cells were set to 1. One sample student's t-test p-PERK: p=0.4425, t=0.9497, df=2. BIP: p= 1.311, t= 2.482, df=2.

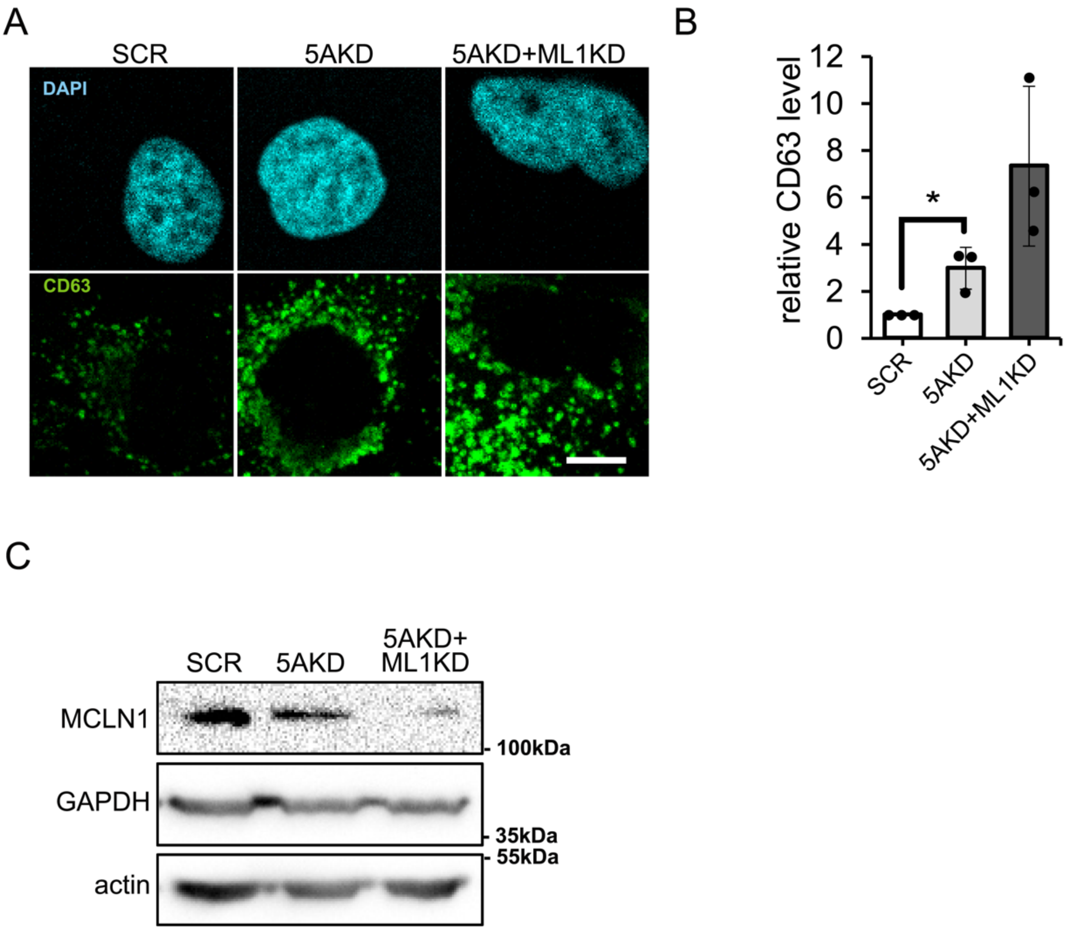

Supplementary figure 4 | Lysosome accumulation in INPP5A-depleted cells is further aggravated by loss of Mucolipin 1. (A) Representative confocal images of fixed HeLa cells treated with control (SCR), INPP5A siRNA (5AKD), or INPP5A and mucolipin 1 siRNA (5AKD+ML1KD) and stained for CD63 (green). Blue, DAPI-stained nuclei. Scale bar, 10 µm. (B) Quantification of organelle levels as exemplified by representative data in A. Unpaired t-test: p=0.018. (C) Immunoblot analysis of HeLa cells treated as in (A). Immunoblots were decorated with antibodies against mucolipin 1 (MCLN1), GAPDH, and β-actin.

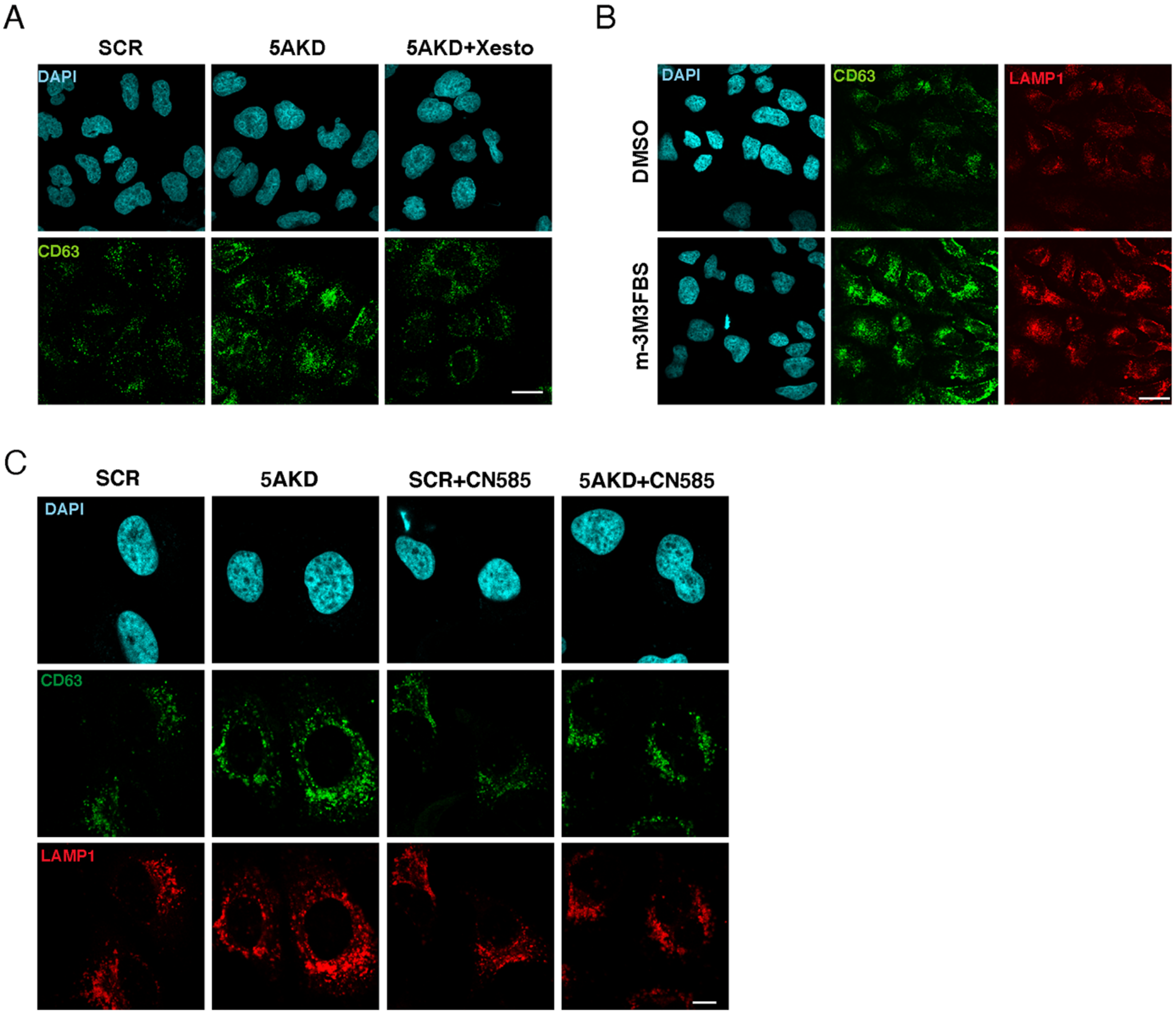

Supplementary figure 5 | Phospholipase C-mediated generation of IP_3 triggers lysosome biogenesis via activation of calcineurin. (A) Representative confocal images of fixed HeLa cells treated with control (SCR) or INPP5A siRNA (5AKD) treated with DMSO (-) or with 1µM Xestospongin C (Xesto) for 18 h and stained for CD63 (green). Blue, DAPI-stained nuclei. Scale bar, 20 µm. (B) Representative confocal images of fixed HeLa cells with DMSO or with 1µM m-3M3-FBS for 24 h and stained with antibodies against CD63 (green) and LAMP1 (red). Blue, DAPI-stained nuclei. Scale bar, 25 µm. (C) Representative confocal images of HeLa cells treated with control (SCR) or INPP5A siRNA (5AKD) treated with DMSO (-) or with 5µM CN585 for 24 h and stained with antibodies against CD63 (green). Blue, DAPI-stained nuclei. Scale bar, 10 µm.

Supplementary table 1 | Oligonucleotides used in this study

siRNA name	Sequence	Souce	Reference
Universal Negative Control #1	n.a	Sigma MISSION	SIC001-10NMOL
INPP5A#1	5'-GCACCGCGCUCUUGGAGUU-3'	Sigma MISSION	Customized
ITPR1	n.a	Dharmacon	L-006207-02-0005
ITPR2	n.a	Dharmacon	L-006208-02-0005
ITPR3	n.a	Dharmacon	L-006209-02-0005
TFEB	n.a	Dharmacon	L-009798-00-0005
TFE3	n.a	Dharmacon	L-009363-00-0005
MCOLN1	n.a	Dharmacon	L-006281-00-0005

Name	Sequence from 5´ to 3'
**ATP6AP1 FW**	CAGCGACTTGCAGCTCTCTAC
**ATP6AP1 Rev**	TGAAATCCTCAATGCTCAGCTTG
**CD63 FW**	CAGTGGTCATCATCGCAGTG
**CD63 Rev**	ATCGAAGCAGTGTGGTTGTTT
**LAMP1 Fw**	TCTCAGTGAACTACGACACCA
**LAMP1 Rev**	AGTGTATGTCCTCTTCCAAAAGC
**ASAH1 Fw**	AGATGTCATGTGGATAGGGTTCC
**ASAH1 Rev**	GGGGCCAATATCTTGGTCTTG
**INPP5A-Fw**	ACACGAACATGGCACTAGGA
**INPP5A-Rev**	GGGCGTGCTCTCTAAGGTAT
**SQSTM1 P62 FW**	GCACCCCAATGTGATCTGC
**SQSTM1 P62 Rev**	CGCTACACAAGTCGTAGTCTGG
**MAP1LC3A FW**	AACATGAGCGAGTTGGTCAAG
**MAP1LC3A Rev**	GCTCGTAGATGTCCGCGAT
**BCN1 FW**	CCATGCAGGTGAGCTTCGT
**BCN1 Rev**	GAATCTGCGAGAGACACCATC

Supplementary table 2 | Antibodies used in this study

Antibody	Species	Dilution		Source	Catalogue number
		ICC	IB		
**Primary antibodies**					
CD63	ms	1:200		Millipore	cbl553
GM130	ms	1:200		BD transduction	610822
LAMP1	Rb	1:200		Cell signaling	9091P
EEA1	ms	1:100		BD transduction	610456
Calreticulin	Rb	1:500		ThermoFisher	PA3-900
b1-Integrin	Ms	1:200		BD transduction	610467
phospho-P70 S6 Kinase T389	Rb		1:1000	Cell signaling	9234
P70 S6 Kinase	Rb		1:1000	Cell signaling	2708
LC3	Rb	1:200	1:1000	Sigma	8918
sqstm1\p62	Ms		1:5000	Abcam	ab56416
BIP	Rb		1:1000	Cell signaling	3177
Phospho-PERK (Thr980)	Rb		1:1000	Cell signaling	3179
PERK	Rb		1:1000	Cell signaling	5683
phospho ERK	Ms		1:5000	Sigma	M8159
ERK	Rb		1:2500	Abcam	ab17942-50
INPP5A	Rb		1:500	ProteinTech	21723-1-AP
MCOLN1	Rb		1:500	Home made in Thomas Jentsch lab MDC/FMP	
TFEB	Ms	1:1000		Santa Cruze	sc-101532
**Secondary antibodies**					
α-ms IgG (H+L) AF488	gt	1:400		Thermo Fisher	A11029
α-rb IgG (H+L) AF488	gt	1:400			A11034
α-rb IgG (H+L) AF647	gt	1:400			A21244
α-ms IgM (H+L) AF568	gt	1:400			A21043
α-rb IRDye680RD IgG(H+L)	gt		1:10000	LI-COR Biosciences	926 -68071
α-rb IRDye800RD IgG(H+L)	gt		1:10000		926 -32211
α-msIRDye680RD IgG(H+L)	gt		1:10000		925 -68070
α-msIRDye800RDIgG(H+L)	gt		1:10000		926 -32210
